# Reading Habits Among Older Adults in Relation to Level and 15-Year Changes in Verbal Fluency and Episodic Recall

**DOI:** 10.3389/fpsyg.2018.01872

**Published:** 2018-09-27

**Authors:** Daniel Eriksson Sörman, Jessica Körning Ljungberg, Michael Rönnlund

**Affiliations:** Department of Psychology, Umeå University, Umeå, Sweden

**Keywords:** reading habits, cognitive aging, longitudinal analyses, verbal fluency, episodic recall, early adult intelligence

## Abstract

The main objective of this study was to investigate reading habits in older adults in relation to level and 15-year changes in verbal fluency and episodic recall. We examined a sample of 1157 participants (≥55 years at baseline) up to 15 years after the baseline assessment using latent growth curve modeling of cognitive measures with baseline reading frequency (books, weekly magazines) as a predictor of cognitive level (intercept) and rate of change (slope). Subgroup analyses were performed to investigate the role of an early adult *g* factor in the association between reading habits and cognitive ability in midlife. Frequent reading of books, but not of magazines, was associated with higher levels of verbal fluency and recall but unrelated to rate of longitudinal decline. Subgroup analyses indicated that the *g* factor in early adulthood predicted reading and cognitive level in midlife and this factor removed the current association between reading habits and level of cognitive ability (both cognitive factors). The results indicate an enduring relationship between book reading and level of cognitive ability across the adult life span and provide little support of the hypothesis that frequent reading protects against late-life cognitive decline. The extent to which book reading promotes cognitive functioning in childhood/youth remains to be demonstrated. Intervention studies may be useful in this regard.

## Introduction

With increased life expectancies, identifying factors that could reduce cognitive decline in old age is of considerable importance. A number of different factors have been suggested to have such an influence, including engagement in mentally stimulating leisure activities (for reviews, see, e.g., Fuchs et al., 2001; Stern and Munn, 2010; Fallahpour et al., 2016).

Beneficial effects of mental stimulation suggested by some of the studies, are in accordance with a cognitive reserve hypothesis, which posits that activity-related stimulation might reduce the risk of cognitive decline and postpone the onset of behavioral changes even in the presence of dementia pathology (Scarmeas and Stern, 2003). The “reserve” concept is often defined as our ability to efficiently use our brain networks and cognitive components (Stern, 2002) and may involve recruiting additional brain regions to compensate for negative brain changes in old age (Tucker and Stern, 2011). The principal point is that if we stimulate the brain sufficiently, we build up cognitive networks that are protective of age-related neural changes (Hultsch et al., 1999; Scarmeas and Stern, 2003). It should be noted that effects of mental stimulation on the reserve is believed to be modifiable, at least to some extent, over the entire life course (Schooler and Mulatu, 2001).

In regard to the potential influence of mentally stimulating activity on cognitive ability and age-related decline in healthy older adults, the review by Hertzog et al. (1999) noted that several studies (e.g., Hultsch et al., 1999) were suggestive of a protective effect of mental activity on subsequent decline. On the other hand, main effects of activity level (i.e., higher levels of cognitive ability in the more active individuals) paired with a lack of age-by-experience interactions were often observed in the literature (Salthouse, 2006). In his review, Salthouse cited evidence of higher cognitive performance in experts versus non-experts (e.g., chess players versus non-players), in those with more versus less mentally demanding occupations (e.g., visuospatial performance in architects versus non-architects; Salthouse et al., 1990) and in individuals with high versus low in global activity level as determined by self-reports (e.g., Salthouse and Mitchell, 1990) but similar magnitudes of age differences across the groups. Such outcomes made Salthouse (2006) suggest that a pattern of preserved differentiation, that is, a persistent advantage of the more “experienced” or mentally active individuals, rather than differential preservation (i.e., reduced rate of cognitive decline in more experienced individuals) may be an equally likely outcome to expect. In favor of the latter outcome, Gow et al. (2014) found that a principal component activity index that included a broad set of activities was associated with higher cognitive ability (a latent factor reflecting digit symbol and digit span) at age 75, but unrelated to rate of cognitive change across a 10-year period (i.e., up to age 85). As noted by the author the difference in baseline cognitive level motivates to consider a potential reversed causal influence, such that individuals who were more active were so, at least in part, because of higher initial cognitive ability level.

Previous studies involving the same study sample have indicated that overall physical activity (Josefsson et al., 2012) as well as general level of leisure activity, especially social activity, may protect against episodic memory decline in old age (Mousavi-Nasab et al., 2014). However, certain leisure activities may be considered particularly interesting to consider as a potential moderator of age-related cognitive changes. The present study focused on book and magazine reading. The sparse amount of literature investigating associations of reading habits specifically (some studies have used reading as part of composite measures of activity level; e.g., Wilson et al., 2002, 2003; Verghese et al., 2006; Kåreholt et al., 2011) is noteworthy, given that this is historically a major source of transferring knowledge, ideas, narratives, and a type of mental activity that is available to most people in modern society. In addition, it is well known that reading, and reading comprehension, activates several cognitive processes (van den Broek and Espin, 2012). More specifically, working memory capacity, and executive functioning, is crucial to maintain information from the text and to continuously update the reader with new information when reading (Baddeley, 2003; Sesma et al., 2009; Swanson and O’Connor, 2009). Furthermore, text processing, together with other information already active in working memory, is believed to trigger the spread of activation through episodic representations of the text and the reader’s semantic knowledge (McKoon and Ratcliff, 1992) and involves activation of the ability of decoding (Perfetti, 1985), reading fluency (Fuchs et al., 2001), selective attention, and comprehension monitoring (Oakhill et al., 2005). Reading may also have an impact on episodic memory by activating the frontal lobe areas in the brain. For example, prefrontal cortex that is known for supervising working memory (Curtis and Esposito, 2003) plays also a crucial role for the episodic memory (e.g., Shallice et al., 1994; Wheeler et al., 1995, 1997), and the left dorsolateral areas as well as the mediotemporal lobe are in particular recruited by both memory systems (Cabeza et al., 2002). These findings have led to the conclusion that the conscious monitoring of incoming information of a transient perceptual representation (working memory) or the output of memory search in episodic memory are both processed in the same areas (Cabeza et al., 2002). These shared brain resources could imply that reading as activity could not only be stimulating working memory but also episodic memory, a system that rarely has been studied in relation to reading.

A limited number of prior studies have considered reading in relation to cognitive ability factors in older adults, and to our knowledge, all of these are suggestive of potential relationship with cognitive functioning in older adults. Gallucci et al. (2009), for example, reported higher performance on a measure of global cognitive functioning (MMSE; Folstein et al., 1975) in older participants who read books (fiction/non-fiction) compared with those who reported reading only newspapers, and lowest performance in those reading neither books nor newspapers. However, a cross-sectional association is, for the reason already mentioned, provide only weak evidence that reading impacts cognition due to lack of knowledge as to whether reading moderates age changes or whether the association reflects a premorbid association (potentially evident already in terms of peak level of cognitive performance). In a related vein, a study by Rice and Meyer (1985) indicated that engagement in reading habits among older adults were paired with higher levels of prose recall, but a similar advantage on the part of those engaged in reading behaviors were also observed in younger adults. This might suggest a persistent association between reading and recall (i.e., a pattern of preserved differentiation) rather than reduced cognitive decline in older readers. Without longitudinal evidence, potential accounts of the findings are hard to separate, though.

Few studies have to our knowledge investigated the possibility that reading might actually moderate rate of cognitive *change*. However, one study with longitudinal follow-up by Wang et al. (2006) concluded that different aspects of reading, calculated as hours spent per week (magazines, newspapers, and books forming a composite measure) were associated with reduced risk of cognitive impairment. Once more, global cognitive functioning (MMSE scores) was assessed, using suggested cutoffs values as markers of cognitive impairment at follow-up. The fact that reading was associated with reduced risk of reaching the threshold values at follow-up could of course be the result of the a preventive influence of reading. However, since it is unclear whether the readers/non-readers were different or similar in terms of mean MMSE score at baseline, an alternative possibility is that the amount of time-related MMSE decline sufficient to reach the threshold values were simply smaller in the non-readers; in other words, a pattern of preserved differentiation might have resulted in the same net outcome as a pattern of reduced decline in those engaged in reading behaviors compared with infrequent readers.

Given the sparse evidence regarding the relationship between reading habits and late-life cognitive changes, the objective of the present study was to examine associations between reading and cognitive level and long-term (15-year) changes in a sample of older adults (55-85 years at baseline). Some prior studies lumped different media together but here two different aspects of reading habits (book reading and weekly magazine reading) were considered. Two separate cognitive abilities functioning were targeted: verbal fluency and episodic recall. Verbal fluency tasks are sometimes used not only to assess executive functioning but also reflect semantic processing ability and word knowledge (McDowd et al., 2011). Presumably, the requirement of rapid retrieval of semantic information required paired with executive demands make verbal fluency measures more age sensitive than measures of semantic knowledge/crystallized intelligence (see Nyberg et al., 2003). Episodic recall may be considered less dependent of acquired knowledge and assumed to reflect our capacity to retain information concerning personally experienced events and underlie our ability to mentally travel in time (Tulving, 1972, 1983) and exhibit a gradual mean level deterioration past age 60 (Rönnlund et al., 2005). As previously noted, both verbal retrieval/fluency and episodic memory functions are obviously activated by reading, and could constitute a type of natural training of these functions.

To control the possibility that late-life associations between reading habits and levels of cognitive functions reflects early adult variations in cognitive ability (cf. Gow et al., 2012), data for a sample of men assessed in midlife with regard to episodic recall, verbal fluency, and reading habits, and for which scores on cognitive tests taken at age 18 were available was examined.

## Materials and Methods

### Study Population

The data emanated from the Betula prospective cohort study (Nilsson et al., 1997; Rönnlund and Nilsson, 2006), a longitudinal study on memory, aging, and health in Umeå, Sweden, that involved stratified randomized sampling (age and sex). Data were collected over six test waves: 1988–1990 (T1), 1993–1995 (T2), 1998–2000 (T3), 2003–2005 (T4), 2008–2010 (T5), and 2013-2014 (T6) and involved assessments of health and cognitive functions (two separate sessions for each test wave; for further details, see Nilsson et al., 1997).

In the main analyses of the present study, data for two samples (Sample 1 and Sample 3) and four waves (T2-T5) were examined, since the questions concerning reading habits were first included at T2 and as these two samples are the only in the Betula study that have been assessed across all test waves after T2. In analyses involving early adult cognitive ability, a subsample of men (in S1 and S3; *n* = 262; age 45-55 years) for which data on three cognitive makers at age 18 had been retrieved from draft boards (see Rönnlund et al., 2015), were examined. There was some overlap with the sample used for the longitudinal analyses (79 of the 55-year-olds were also included in the first sample).

### Participants

In total, 1227 participants aged 55 years or older responded to the questions about reading books and/or magazines at baseline (T2). Participants also responded to how often they read the daily newspaper. However, 96.7% of the participants reported that they did this on a daily basis, and thus, this variable was not included in the analyses due to extreme skew in the data. A few participants with missing data on the cognitive tasks (*n* = 52), and education (*n* = 7) were excluded. In addition, a few participants (*n* = 11) with progress of dementia at inclusion were discarded (retrospective analyses performed by a geriatric psychiatrist concluded that some participants had ongoing progress of dementia at T2). Thus, data for 1157 participants were included in the main analyses. The sample included 498 men and 659 women from Sample 1 (*n* = 537) and 3 (*n* = 620). Mean age was 68.8 years (*SD* = 9.7) and the mean for years of education was 8.66 (*SD* = 3.4). Cognitive data were available for 878, 656, and 370 participants at the 5-, 10-, and 15-year follow-up, respectively.

In the subsample with early adult cognitive data, all 262 participants had responded to questions regarding reading at baseline. Mean baseline age in this sample was 49.9 years (*SD* = 4.0). Mean years of education was 12.2 (*SD* = 3.9).

### Measures

#### Book Reading

The question about how often participants read books was part of a leisure activity questionnaire (16 items) requesting the participants to specify frequency of each activity on a five-point scale: “never,” “occasionally,” “a few times a month,” “sometimes per week,” “and every day.” In the main analyses, we dichotomized the variable into “Infrequent book readers” (never – few times a month, coded as 0) and “Frequent book readers” (sometimes per week – daily, coded as 1). The cutoff was motivated by the distinct difference of doing an activity on a regular basis, compared to more sporadic and yielded fairly equal group sizes (cf. Section “Results”). This grouping of participants into low-high frequency of book reading constituted the base of the analyses. However, we also aimed to perform additional analyses treating frequency of book reading as a continuous variable although results from these additional analyses must be taken with caution since frequency of reading was rated on an ordinal scale and structural equation modeling (SEM) is a regression-based approach based on linear relationships.

#### Reading of Weekly Magazines

As for book reading, participants rated frequency of reading weekly magazines as a part of the Leisure activity questionnaire. Similarly, we dichotomized the variable into “Infrequent magazine readers” (never – few times a month, coded as 0) and “Frequent magazine readers” (sometimes per week – daily, coded as 1).

#### Episodic Recall

Five tasks were used as measures of episodic recall (for a more detailed description, see Rönnlund and Nilsson, 2006): (1) sentence recall (SR): free oral recall of 16 sentences (e.g., scratch your eye) encoded without enactment. (2) Action recall (AR): free oral recall of 16 verb-noun sentences (e.g., point at the catalog) that were enacted by the participant. For both SR and AR, a metronome beep at that rate of 8 s/item indicated the time for each stimuli to be presented. The time limit for recall was 2 min. (3) Category-cued recall of nouns from the sentence list. (4) Category-cued recall of nouns from the list of actions. For cued recall, a sheet with semantic categories referring to the nouns presented in the SR and AR tasks were used as cues. The time limit for recall was 3 min. (5) Free recall of 12 words (nouns) presented at a pace of 2 s, time for recall was 45 s. A manifest variable, based on *z*-transformations of performance in each task, was created for each test wave. In latent variable analyses, the number of recalled items in the action (AR; i.e., free + cued recall) and SR conditions (SR; free + cued recall) served as separate indicators of a recall factor together with word recall (WR).

#### Verbal Fluency

Three measures (cf. Nyberg et al., 2003) that required verbal generation of as many words as possible (except names) during 1 min were used: (A) words beginning with the letter A [fluency A (FLA)], (B) words with the initial letter M and containing five letters [fluency M (FLM)], and (C) professions with initial letter B [fluency B (FLB)]. Maximum score for each task was the number of correctly generated words. A manifest variable, based on *z*-transformations of performance in each fluency task based on M/SD of the baseline sample, was created for each test wave.

#### Early Adult g

Three cognitive measures in the Swedish Enlistment Battery (SEB) used during the years 1954–1967 were used as indicators of an early adult g factor. Instructions (INS), intended to measure the primary factor induction. INS contained verbal INS to make markings on the answer sheet, so as to fulfill the conditions specified by the INS [e.g., “Mark the first letter of the second word in the present sentence” (correct answer: t)]. Concept discrimination (CD) involved classification of words and technical comprehension (TC) involved a set of illustrated technical and physical problems (Carlstedt, 2000, unpublished). For each test, performance was recorded in the form of stanine scores (*M* = 5, *SD* = 2). A g factor reflecting the foregoing measures was found to be a strong predictor of g and working memory capacity in late adulthood (Rönnlund et al., 2015).

#### Covariates

Covariates included in the analyses were age, sex (female coded as 0, male coded as 1), and years of formal education.

### Statistical Analysis

We tested baseline differences in participant characteristics between frequent/infrequent readers *via* Student’s *t*-tests and chi-square analyses. To investigate associations with level and longitudinal changes in cognitive performance, latent growth models were employed. Data were analyzed with SPSS-23 and AMOS-23 using full information maximum likelihood (FIML) estimation. Performance in episodic recall and verbal fluency were used as the dependent variables in separate models, including four waves of cognitive data. First, the fit of the unconditional models including the four waves of cognitive data (separately for episodic recall and semantic fluency) were tested to see whether means and variances for the intercept and slope motivates inclusion of additional variables. Next, the fit of conditional models were tested with book reading, weekly magazine reading, age, sex, and years of education as simultaneous predictors of intercept and slope.

We used two fit indices to evaluate model fit: Bentler’s comparative fit index (CFI) and the root mean square error of approximation (RMSEA). For CFI, values equal or greater than 0.95 is warranted for acceptable fit (Hu and Bentler, 1999). For RMSEA, a value of 0.06 or less is indicative of good model fit and a value of 0.08 indicating reasonable fit (Browne and Cudeck, 1993; Hu and Bentler, 1999).

## Results

Of the 1157 participants, 42.5% were frequently reading books (sometimes per week - every day), whereas 57.5% of the participants were infrequent book readers (never - few times a month). As regards reading magazines, 53.5% of the participants were classified as frequent readers whereas 46.5% were infrequent readers. Participant characteristics across the resulting groups, are provided in **Table 1**. Results from a Student’s *t*-test analyses showed that frequent book readers had more years of education and chi-square analysis revealed that frequent readers more often were females, but no difference with regard to age was observed. Moreover, frequent book readers performed better in episodic recall and verbal fluency at baseline. With regard to weekly magazines, results showed that frequent readers were older, more often females, and had fewer years of formal education. They also performed worse in both cognitive domains at baseline. This relationship between frequent magazine reading and cognitive performance in episodic recall and verbal fluency was opposite that of the frequent book reading and cognitive performance. Thus, the next step was to investigate if these relationships would persist following the adjustment of covariates in structural equation models.

**Table 1:** Baseline characteristics of the study sample (*n* = 1157).

	Frequent book readers (42.5%)			Infrequent book readers (57.5%)		
	Mean	*SD*	%	Mean	*SD*	%
Age	68.93	9.6		68.68	9.7	
Females			64.4			51.4^∗∗∗^
Years of education	9.76	3.9		7.84^∗∗∗^	2.7	
Episodic recall – *z* score	0.22	1.0		–0.16^∗∗∗^	0.9	
Verbal fluency – *z* score	0.20	1.0		–0.15^∗∗∗^	1.0	

	**Frequent magazine readers (53.5%)**			**Infrequent magazine readers (46.5%)**		
	**Mean**	***SD***	**%**	**Mean**	***SD***	**%**

Age	69.63	9.6		67.66^∗∗∗^	9.7	
Females			65.0			47.5^∗∗∗^
Years of education	8.30	3.3		9.07^∗∗∗^	3.6	
Episodic recall – *z* score	–0.07	1.0		0.10^∗∗^	0.9	
Verbal fluency – *z* score	–0.07	0.9		0.09^∗∗^	0.9	

*^∗∗^*p* < 0.01, ^∗∗∗^*p* < 0.001.*

Next, the unconditional latent growth curve models were analyzed, separately for episodic recall and verbal fluency. For CFI, all models yielded values that were equal or greater than 0.95. For RMSEA, the two models with verbal fluency (0.09) and episodic recall (0.07) showed values slightly over suggested cutoff (0.06). The variances were significant for intercept (performance at baseline) in all models (*p* < 0.001), indicating that there are meaningful between-person differences in level of performance across measures. More importantly, the variances in slope were also significant, for both verbal fluency (*p* = 0.02) and episodic recall (*p* < 0.001), indicating meaningful differences between participants with regard to change over time. For both episodic recall (*p* = 0.02) and verbal fluency (*p* < 0.001), baseline performance correlated with slope which indicated that initial status was related changes over time. In conclusion, further analyses were motivated by investigating if intercept (level of performance) and slope (time-related change) differ dependent on how often the participants were engaged in reading.

**FIGURE 1:**
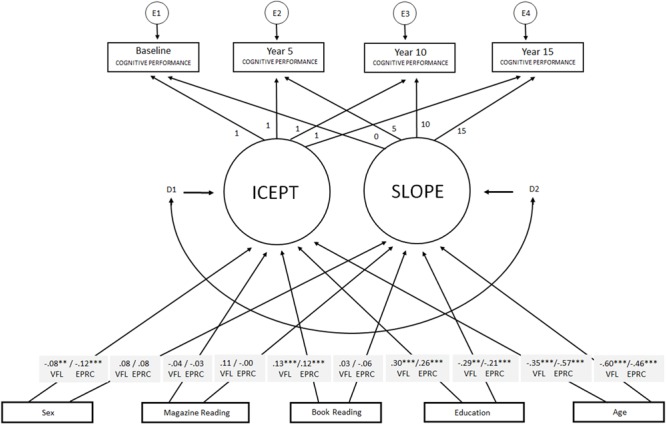
The latent growth curve model/s used in the present study. Book reading, magazine reading, age, sex, and years of formal education were used as covariates of intercept and slope. Manifest variables of cognitive performance were used at each test wave. Predictors were correlated by double headed arrows in the model. Standardized regression weights between each predictor variable and intercept and slope are highlighted in gray. ICEPT, intercept; VFL, verbal fluency; EPRC, episodic recall. ^∗∗^*p* < 0.01, ^∗∗∗^*p* < 0.001.

Next, conditional models including age, sex, years of education, weekly magazine reading, and book reading were tested (**Figure 1**). The results are displayed in **Table 2**.

**Table 2:** Fit indices for conditional models of the reading-cognitive performance relationship.

				→ ICEPT				→ SLOPE			
				Means		Variance		Means		Variance	
Model	CFI	RMSEA	χ*^2^/df*	Estimate	*P*	Estimate	*P*	Estimate	*P*	Estimate	*P*
Verbal fluency	0.96	0.07	5.90	1.55 (0.22)	<0.001	0.47 (0.03)	<0.001	0.07 (0.02)	<0.001	0.00 (0.00)	0.06
Episodic recall	0.98	0.05	4.22	3.34 (0.20)	<0.001	0.38 (0.02)	<0.001	0.09 (0.02)	<0.001	0.01 (0.00)	<0.001

*Models includes age, sex, years of education, and book reading as covariates.*

Values for CFI (≥0.95) indicated good fit of both models. In addition, episodic recall had a RMSEA value of 0.05, which confirm these results, and for verbal fluency, the RMSEA was indicative of reasonable fit (<0.07). With regard to slope, variance in episodic recall was still significant after the inclusion of covariates (*p* < 0.001), and almost for verbal fluency (*p* = 0.06). Means were significant for both intercept and slope in all models (*p* < 0.001).

Finally, straight associations between predictor variables and cognitive performance were investigated. The results are presented in **Table 3**.

**Table 3:** Regression weights of predictors in the conditional latent growth curve models that include age, sex, years of education, magazine, and book reading as covariates.

	Verbal fluency		Episodic recall	
	β1	*P*	β1	*P*
Book reading → I	0.13	<0.001	0.12	<0.001
Book reading → S	0.03	0.78	–0.06	0.27
Magazine reading → I	–0.04	0.09	–0.03	0.24
Magazine reading → S	0.11	0.22	–0.00	0.96
Age → I	–0.35	<0.001	–0.57	<0.001
Age → S	–0.60	<0.001	–0.46	<0.001
Male → I	–0.08	0.004	–0.12	<0.001
Male → S	0.08	0.36	0.08	0.13
Education → I	0.30	<0.001	0.26	<0.001
Education → S	–0.29	0.004	–0.21	<0.001

*I, intercept; S, slope; β1; standardized regression weight.*

Frequent books readers (once a week or more) had significantly higher intercept (baseline performance) in verbal fluency (standardized β = 0.13, S.E. = 0.05, *p* < 0.001), but there was no difference with regard to slope (β = 0.03, S.E. = 0.00, *p* = 0.78). Reading of magazines was not related to intercept in verbal fluency (β = -0.04, S.E. = 0.05, *p* = 0.09) nor slope (β = 0.11, S.E. = 0.00, *p* = 0.22).

With regard to episodic recall, frequent book reading was associated with a higher intercept (β = 0.12, S.E. = 0.04, *p* < 0.001), but book reading was unrelated to slope (β = -0.06, S.E. = 0.00, *p* = 0.27). As before no relation to intercept (β = -0.03, S.E. = 0.04, *p* = 0.24) or slope (β = -0.00, S.E. = 0.00, *p* = 0.96) was observed for magazine reading.

Among the other predictors, higher age was significantly related to both lower intercept and a more negative change over time, for both cognitive abilities. Being female was associated with higher level of both episodic recall and verbal fluency, but sex was unrelated to slope. Longer education was associated with higher levels of recall and fluency but more negative change over the follow-up period.

Analyses treating frequency of book reading and weekly magazine reading as continuous variables, revealed similar patterns of results; more frequent book reading was associated with higher intercept but not with slope. No associations were found for reading weekly magazines. Also, to rule out the possibility associations with the reading differs if using older age groups, we performed additional analyses only including participants 65 years or older at baseline. However, book reading was not more protective for this specific age group, i.e., reading level was still related to intercept, but not to slope. For complementary analyses in which participants reading habits of both magazines and books were combined.^1^

### Reading and Cognitive Factors in Midlife in Relation to an Early Adult g Factor

**FIGURE 2:**
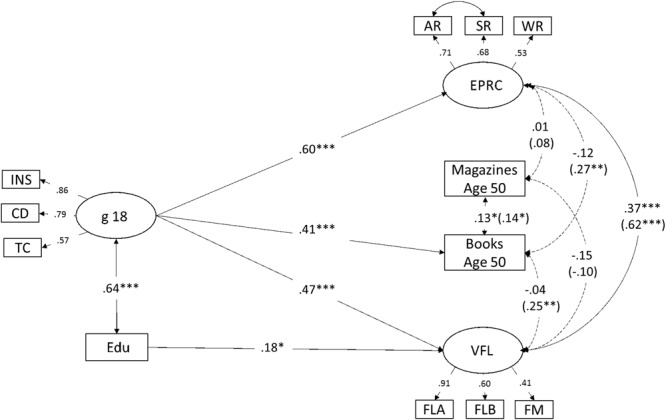
Structural model of the relationship between an age 18 general ability (g 18) factor and education and reading habits (magazines, book) and levels of cognitive factors in midlife (mean age 50). Regression weights are standardized coefficients (βs). Non-significant paths from g 18 and education to the midlife measures (i.e., reading, cognitive factors) were omitted. Dashed lines indicate non-significant associations between midlife factors after the adjustment of age 18 general ability (g 18). Standardized coefficients (βs) before the inclusion of g into the model are presented in brackets. INS, instructions; CD, concept discrimination; TC, technical comprehension; g 18, g at age 18; Edu, education; EPRC, episodic recall; AR, action recall; SR, sentence recall; WR, word recall; VFL, verbal fluency; FLA, fluency A; FLB, fluency B; FLM, fluency M; ^∗^*p* < 0.05, ^∗∗^*p* < 0.01, ^∗∗∗^*p* < 0.001.

To examine the association between early adult g on reading, level of the cognitive factors, and on the relationship between the former factors (i.e., after versus before consideration of an early adult g factor), a structural model involving g (age 18) and education as a predictor of latent cognitive (verbal fluency, episodic recall) factors and reading (magazines, books) in midlife was tested. The model fit was good as judged by the two fit indices (CFI = 0.986, RMSEA = 0.036) and non-significant in terms of a χ^2^ goodness-of-fit test (*df* = 41) = 55.6, *p* = 0.08. The model, including values for significant standardized regression coefficients (β-values) are summarized in **Figure 2**.

The g factor was strongly predictive not only of episodic recall and fluency (βs 0.47-0.60) but also of future book reading (but not reading of weakly magazines). In addition, as compared with a model including the midlife cognitive factors only (values in brackets), the inclusion of the g factor eliminated the association between midlife book reading and cognitive factors. Finally, years of education was a unique predictor of verbal fluency, but not of episodic recall.

## Discussion

Results of latent growth curve analyses, including four test waves of cognitive measurements, showed that participants that read books exhibited higher level of performances in both verbal fluency and episodic recall in models including age, years of education, and sex as the covariates. By contrast, reading level was unrelated to slope. In other words, we observed a cognitive advantage on the part of those who read more frequently, whereas the rate of age-related decline in episodic recall or verbal fluency was unrelated to readings, hence demonstrating a pattern of preserved differentiation of frequent/infrequent book readers in regard to verbal fluency and episodic memory recall.

The finding of an association between book reading and cognitive ability level is consistent with findings in prior studies (e.g., Gallucci et al., 2009). Since reading magazines exhibited no relationship with any of the cognitive abilities, the practice of combining reading of books and magazines as in some previous studies is questionable. Reading magazines may not represent the same type of in-depth reading activity as that associated with books. Given the nature of magazines consisting of many pictures and short articles, it is possible that such activity may be more superficial and less likely to engage cognitive resources. Hence, a baseline difference in cognitive level could indicate that book reading, but not magazine reading, boosted cognitive level prior to baseline. Using the terms by Christensen et al. (1997), reading might be proposed to be compensatory of neurodegenerative changes with aging, rather than protective (i.e., slowing rate of decline).

The notion of being compensatory warrants consideration of the timing (and directionality) of the proposed influence. In this regard, the present study benefitted from a rare opportunity to control for early adult intelligence in a subsample of men for which conscript data were available. The results of these analyses were informative to the extent that the associations between the targeted cognitive functions (episodic recall and verbal fluency) and book reading was eliminated once the early adult general ability factor was taken into account. Indeed, level of early adult intelligence appeared to account not only for a sizeable portion of variance in midlife level of episodic recall and verbal fluency but was also a good predictor of reading habits around 32 years later. These aspects of the present results are in agreement with Gow et al. (2012) who reported a similar pattern for a principal component reflecting “socio-intellectual activity” (e.g., “visiting friends and family” and “reading a book”) in relation to general cognitive ability, processing speed and memory, namely, significant activity-cognition associations at age 70 that were eliminated once childhood IQ (at age 11) was adjusted for. Thus, the present results indicate a patterns of an enduring cognitive difference between those who read more or less frequently in old age, differences that may reflect early adult differences in cognitive ability rather than differences in reading habits. It is important to note though that we only had information on early adult intelligence among 79 of the participants in the subsample that also were included also in the longitudinal analyses. Thus, even if our interpretation of the results seem reasonable, a greater overlap between samples would have been warranted to be able to determine a causal link between early intelligence and reading patterns later in life.

The association of reading with early adult g could in principle be the result of common genetic factors, but, obviously, reading in childhood and youth could boost peak level cognitive performance given that brain plasticity is greater among children and adolescents and thereby influence late-life cognitive level, a possibility that needs to be considered in future studies. It is also important to note that the present patterns of findings do not exclude the possibility that high intensive reading in old age may benefit certain cognitive processes. Findings obtained in an intervention study by Tesky et al. (2011) showed that when participants in older age groups increased their amount of engagement in mental activities such as for example reading, the speed of information processing were improved. Reduced subjective memory decline was also found in participants between the ages of 60 and 75 years. However, since the focus of interest in this study was on a general increase of engagement, grouping many different leisure activities together, the impact of increased reading solely is hard to predict. Against the findings in the present study, it is finally important to take into consideration that, apart from stimulating semantic knowledge, frequently reading books (i.e., novels) may benefit other processes than the ones investigated here, for example, perspective taking/empathy (Bal and Veltkamp, 2013) and may be an effective means to reduce mental distress (Nell, 2004).

Interestingly, another predictor included in the analyses, education, often considered as a major component contributing to the cognitive reserve (Stern, 2002), revealed a different impact on the outcome variables compared to book reading. In our study, although positively associated with intercept for both cognitive measures (except in the analyses involving a g factor in early adulthood), more years of education was also related to more rapid cognitive decline. Although speculative, these findings may have the following implications: (1) later in life, after retirement, the loss of cognitive stimulation may cause a reduction in cognitive functioning (e.g., Christensen et al., 1996; Schooler et al., 1999). Individuals with more years of education most likely have had occupations with higher demands on cognitive functioning and inactivity after retirement may have decreased cognitive stimulation and thus speeded up the cognitive aging process (e.g., Finkel et al., 2009). (2) Even though brain training through book reading in older age groups could not be concluded to be a protective factor against memory degradation in the present study other studies argues that to maintain cognitive functioning in old age, a use dependency perspective might be applicable, suggesting that living a cognitively stimulating life after retirement is important to maintain good cognition in old age (Almond, 2010). In regard to education, it might finally be noted that it was predictive of midlife level of verbal fluency (but not episodic recall) over and beyond the g factor, which seems consistent with some influence on midlife level of cognitive performance at least.

In interpreting our results, some limitations should be noted. First, our measure of reading habits is a rather rough measure. We separated participants based on times per month undertaken this activity, but still we do not know how many hours, for instance, were spent on reading. Such information would have improved the reliability of our measure. Second, we did not have any information on what type of literature, or how cognitively challenging books or magazines, participants usually engaged in. Most likely persons read what they find interesting based on interest and/or curiosity, and adapts the difficulty level after “current state.” However, it is plausible that literature perceived as cognitively demanding to a greater extent promotes enrichment that in turn would be beneficial for the cognitive abilities of interest in the present study.

## Conclusion and Further Research

Results from this study provided little support for the hypothesis reading affects the rate of age-related decline in episodic recall and verbal fluency. Frequent book readers performed better that infrequent book readers both at baseline and across time (15 years), with a stable cognitive advantage over the follow-up period. This pattern of preserved differentiation might suggest that the passage of a certain threshold for cognitive impairment occurs a later time point in frequent book readers. Still, it is important to note that analyses of conscript data indicated that early adult intelligence accounted not only for a large variance in midlife level of episodic recall and verbal fluency but also predicted to a great extent reading habits around 32 years later and, when accounted for, removed the association between reading habits and the two cognitive factors. Thus, a late-life association of reading and health outcomes might reflect other facts than book reading *per se*.

Future research in the field may benefit from a deeper understanding of the impact of the demands of the book and the required cognitive underpinnings, for example, whether the preferred literature are novels or non-fiction, whether the books are written in the readers’ mother tongue or a second language. Future research should include more refined measures of book reading and also examine if patterns can be found across other activated cognitive processes than those investigated in the present study. Intervention studies is also needed to fully examine the causal influence of book reading on cognitive functioning. Finally, the possibility that book reading in childhood/youth has a profound impact on level of cognitive functioning that may persist through the life course needs to be evaluated.

## Ethics Statement

This study was approved by the Ethics Review Board, Umeå University. All subjects gave written informed consent in accordance with the Declaration of Helsinki.

## Author Contributions

DS, JL, and MR developed the research questions and wrote the Introduction, Materials and Methods, Results, and the Conclusion sections. DS and MR performed the formal analyses. All authors have contributed equally.

## Conflict of Interest Statement

The authors declare that the research was conducted in the absence of any commercial or financial relationships that could be construed as a potential conflict of interest.

## Footnotes

1 Mixed analyses of variance (ANOVAs) were also performed in which groups were created based on reading habits of both magazines and books (*n* = 360; high frequency on both books and magazines = 89; high frequency on books but low on magazines = 78; low frequency on books but high on magazines = 94 for episodic/95 for fluency; low frequency on both books and magazines = 99). We performed separate 4 (Group) × 4 (Measurement Occasion) analyses with repeated measures on the last factor. Analyses were adjusted for covariates age, gender, and years of education. For episodic recall, test of between subject effects revealed a significant main effect of group; *F*(3,353) = 3.24, MSE = 0.26, *p* = 0.022, $\eta_{p}^{2}$ = 0.027. Pairwise comparisons of groups revealed a significant difference between high frequency on both books and magazines compared to low level on books but high on magazines (*p* = 0.01) and almost significant toward low frequency on both books and magazines (*p* = 0.07). There was, however, no tendency of difference between high frequency on both books and magazines compared to high level on books but low on magazines (*p* = 0.92). For verbal fluency, a similar pattern was found. Test of between subject effects showed a significant main effect of group; *F*(3,354) = 7.54, MSE = 0.41, *p* < 0.001, $\eta_{p}^{2}$ = 0.060. Pairwise comparisons demonstrated a significant difference between high frequency on both books and magazines compared to low frequency on both books and magazines (*p* = 0.02) and compared to low frequency on books but high on magazines (*p* < 0.001), but no difference with high frequency on books and low on magazines (*p* = 0.41). Thus, as in the main analyses, results indicate that it is higher level of book reading, independent of level of magazine reading, that to some extent can be related to greater performance in episodic recall and verbal fluency. The was little support that high frequency of magazine reading is beneficial as compared to low frequency of magazine reading. No interaction between group and time was found for either episodic recall or verbal fluency. Thus, as in the main analyses, groups were similar with regard to change over the 15-year period.
